# Monte Carlo simulation of physical dose enhancement in core-shell magnetic gold nanoparticles with TOPAS

**DOI:** 10.3389/fonc.2022.992358

**Published:** 2022-09-14

**Authors:** Xiaohan Xu, Jianan Wu, Zhitao Dai, Rui Hu, Yaoqin Xie, Luhua Wang

**Affiliations:** ^1^ Department of Radiation Oncology, National Cancer Center/National Clinical Research Center for Cancer/Cancer Hospital & Shenzhen Hospital, Chinese Academy of Medical Sciences and Peking Union Medical College, Shenzhen, China; ^2^ Institute of Biomedical and Health Engineering, Shenzhen Institutes of Advanced Technology, Chinese Academy of Sciences, Shenzhen, China; ^3^ Department of Radiation Oncology, National Cancer Center/National Clinical Research Center for Cancer/Cancer Hospital, Chinese Academy of Medical Sciences and Peking Union Medical College, Beijing, China; ^4^ Department of Radiation Oncology, Affiliated Suzhou Hospital of Nanjing Medical University Suzhou Municipal Hospital, Suzhou, China

**Keywords:** radiotherapy, magnetic gold nanoparticle, dose enhancement factor, magnetic field, TOPAS

## Abstract

The application of metal nanoparticles (MNPs) as sensitization materials is a common strategy that is used to study dose enhancement in radiotherapy. Recent *in vitro* tests have revealed that magnetic gold nanoparticles (NPs) can be used in cancer therapy under a magnetic field to enhance the synergistic efficiency in radiotherapy and photothermal therapy. However, magnetic gold NPs have rarely been studied as sensitization materials. In this study, we obtained further results of the sensitization properties of the magnetic gold NPs (Fe_3_O_4_@AuNPs) with or without magnetic field using the TOPAS-nBio Monte Carlo (MC) toolkit. We analyzed the properties of Fe_3_O_4_@AuNP in a single NP model and in a cell model under monoenergetic photons and brachytherapy, and we investigated whether the magnetic field contributes to the physical sensitization process. Our results revealed that the dose enhancement factor (DEF) of Fe_3_O_4_@AuNPs was lower than that of gold nanoparticles (AuNPs) in a single NP and in a cell irradiated by monoenergetic photons. But it’s worth mentioning that under a magnetic field, the DEF of targeted Fe_3_O_4_@AuNPs in a cell model with a clinical brachytherapy source was 22.17% (cytoplasm) and 6.89% (nucleus) higher than those of AuNPs (50 mg/mL). The Fe_3_O_4_@AuNPs were proved as an effective sensitization materials when combined with the magnetic field in MC simulation for the first time, which contributes to the research on *in vitro* tests on radiosensitization as well as clinical research in future.

## 1 Introduction

Cancer is a serious disease that continues to threaten human health. At present, more than 50% of cancer patients have been treated by radiotherapy (1, 2). Although radiotherapy can kill tumor cells, it simultaneously threatens healthy tissues. Therefore, simulation studies on improving the sensitivity of tumor cells to radiotherapy and minimizing the mortality of healthy cells to enhance the efficiency of radiotherapy can provide a theoretical basis for promoting the clinical application of radiotherapy.

With the rapid developments in biotechnology and nanotechnology (3–5), the use of nanomaterials as radiosensitization materials offers new possibilities for cancer radiotherapy (6–10). NPs prefer to congregate in tumors as a result of enhanced permeability and retention (EPR) (11, 12). In radiotherapy, high atomic number (Z) materials can be used to enhance the dose in tumors in combination with the EPR property (13). AuNPs have exhibited a high X-ray cross section, low toxicity, good biocompatibility, and easy synthesis, thereby attracting significant attention in research on the radiation sensitization of nanomaterials in recent years (14–16). AuNPs have the potential to be applied to medical imaging, medical drug delivery, photothermal therapy, and radiation sensitization therapy. In 2004, Hainfeld et al. demonstrated the radiation dose enhancement effect of AuNPs through animal experiments, which laid the foundation for research on AuNPs in radiation sensitization (17).

Recent advances have revealed the high potential of targeted magnetic NPs in radiotherapy (18), whereby a magnetite core combined with a suitable coating can be bestowed with biochemical and drug-delivery properties (19). For this reason, a magnetite core combined with a gold shell was proposed to improve the stabilization, biocompatibility, and surface reactivity of sensitizing NPs (20).

The mechanisms by which MNPs provide radiosensitization can be divided into three stages, physical, chemical and biological, according to their relative time scale. In the physical stage, the biomolecules are damaged by the secondary electrons, mainly generated by the photoelectric effect. In the chemical stage, the electronically active surface of MNPs catalyzes radical production to damage the DNA, and the very low energy electrons (LEEs) increase the chemical sensitization of DNA to irradiation damage is due to the transient negative ions produced by LEE weaken the bonds within DNA (13). In the biological stage, MNPs increase the sensitization through cell cycle distribution, oxidative stress and DNA repair inhibition (20). In this study, we focused on the physical sensitization, given that data analysis and modeling of NP-induced chemical and biological sensitization remain limited, and their exact mechanisms are poorly understood than physical sensitization (21). This work provides a foundation for further research on chemical and biological sensitization.

The MC method is a computational approach that represents physical processes by simulating numerous random particles (22–24). Commonly used MC codes include Geant4, MCNP, and Fluka, which have a high calculation efficiency. Among these, the Geant4-DNA extension package can be used to simulate the interaction of eV energy electrons. This package has attracted the attention of medical physicists owing to its user-friendly operation interface in the form of TOPAS (25, 26). TOPAS-nBio is an extension of TOPAS that is based on and extends the Geant4 Simulation Toolkit for radiobiology applications (27–29).

With the development of MR-Linacs, there is a clinical need to study the efficacy of magnetic AuNPs to combine and improve diagnosis and radiotherapy. Nevertheless, magnetic AuNPs have rarely been studied as sensitization materials, with or without a magnetic field. To address this limitation, in this study, we used TOPAS and TOPAS-nBio to study the Fe_3_O_4_@AuNP properties in radiotherapy sensitization compared to an AuNP in a single NP and a cell model using monoenergetic photons. Subsequently, we combined the simulation with a magnetic field to investigate the influence on the sensitivity process. Finally, we changed the photon beams with a brachytherapy source to perform the same process. Our work contributes to the research on Fe_3_O_4_@AuNPs in radiotherapy using the MC method and provides a reference for clinical research.

## 2 Materials and methods

### 2.1 Cell uptake of Fe_3_O_4_ @AuNPs by HeLa cells with or without a magnetic field

**Figure 1:**
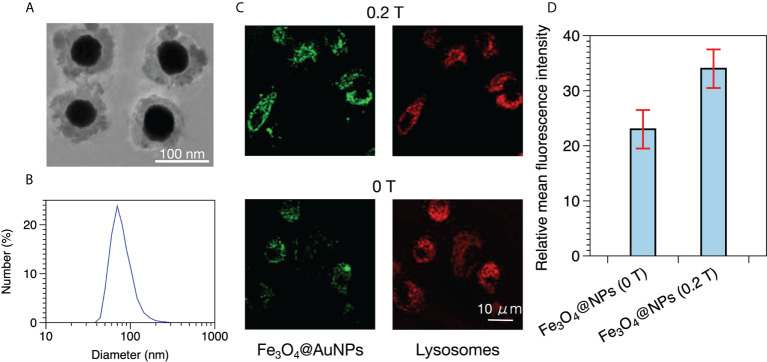
**(A)** TEM image of Fe_3_O_4_@AuNPs. **(B)** Dynamic light scattering analysis of Fe_3_O_4_@AuNPs. **(C)** Distribution of Fe_3_O_4_@AuNPs in HeLa cells with and without a magnetic field in CLSM after 3 h of incubation. **(D)** Fluorescence intensity of Fe_3_O_4_@AuNPs with and without a magnetic field.

The Fe_3_O_4_@AuNP used by Hu et al. consisted of a Fe_3_O_4_ core and a gold shell, as shown in **Figure 1A** (30). The mean diameter of the Fe_3_O_4_@AuNPs was 100 nm according to dynamic light scattering analysis, as illustrated in **Figure 1B**. According to **Figures 1A, B**, we determined that the Fe_3_O_4_@AuNP consisted of a 60 nm diameter Fe_3_O_4_ core and a 20 nm thickness gold shell. Therefore, we selected 100 nm as the diameter of the Fe_3_O_4_@AuNP and used the same Fe_3_O_4_ and Au ratio in our simulation work. Hu used confocal laser scanning microscopy (CLSM) to observe the distribution of the Fe_3_O_4_@AuNPs internalized by the HeLa cells, as depicted in **Figure 1C**. The Fe_3_O_4_@AuNPs and lysosomes were labeled by fluorescein isothiocyanate and LysoTracker Red, and exhibited green and red fluorescence, respectively, in the CLSM. The distributions of the Fe_3_O_4_@AuNPs and lysosomes were clearly partially overlapped, meaning the Fe_3_O_4_@AuNPs were internalized by cells and could be swallowed by the lysosomes in the cytoplasm. We also observed that the green fluorescence intensity with a 0.2 T dipole magnetic field was higher than that without a magnetic field. Hu used flow cytometry to analyze the mean fluorescence intensity to compare the cell uptake of Fe_3_O_4_@AuNPs with and without a magnetic field quantitatively, as illustrated in **Figure 1D**. The results demonstrated that the fluorescence intensity of the Fe_3_O_4_@AuNPs in an external magnetic field was 1.48 times higher than that without a magnetic field. Moreover, Hu demonstrated that Fe_3_O_4_@AuNPs can be used to decrease the viability of HeLa cells in radiotherapy with an external magnetic field (0.2 T). The results indicated that the cell viability was affected by the magnetic field owing to the cell uptake properties being enhanced under the magnetic field. In fact, the cell viability may be affected by the cell uptake properties, the physical dose enhancement of Fe_3_O_4_@AuNPs, and other conditions. In our research, we studied the physical dose enhancement properties of Fe_3_O_4_@AuNPs, with and without a magnetic field, using TOPAS and TOPAS-nBio.

### 2.2 Calculation methods for dose enhancement factor: Two-step and one-step methods

Two methods are used for calculating the DEF with TOPAS and TOPAS-nBio. Lin et al. investigated the dose enhancement of proton and photon irradiation on AuNPs using TOPAS (31). Lin calculated the dose distribution around a single AuNP that changed with the distance from the particle surface and obtained the distribution of the DEF at different distances from the AuNP surface. DEF is defined as the ratio of the deposited dose, with and without the MNP, in water. As the Geant4-DNA physics list is workable in water only, this package cannot be used to calculate the tracks in AuNP accurately; thus, Lin divided the dose calculation into two steps. First, the interaction of a photon beam with an AuNP sphere is modeled with Penelope physics list to obtain the phase space distribution (positions and velocities) of secondary electrons emitted from AuNP surface. Second, the dose distribution of the secondary electrons in a water box is calculated with Geant4-DNA physics list by simulating an electron source with the same phase space distribution from the first step. For simplicity, such a method of calculating the DEF is referred to as the “two-step method” in our research. However, the surface dose distribution around a single AuNP is not the exclusive factor affecting the cell livability, and the effects of the radiation emerging or scattering from an AuNP on the other AuNPs in a cell model should also be considered.

Scientists developed TOPAS-nBio to simulate radiobiological experiments on nanometer scale cells considering the physics, chemistry, and biology effects. TOPAS-nBio supports the assignment of different physical models to different geometry components. Rudek et al. established the AuNPs that were internalized in a cell model irradiated by photons, protons, and carbon ions, respectively, using TOPAS-nBio (32). To define suitable physical modules in different regions, Rudek set the Livermore physics list in the AuNP region and the Geant4-DNA physics list outside the AuNP region. Thereafter, the DEFs in the cytoplasm and nucleus were calculated. This method of calculating the DEF is referred to as the “one-step method” in this work for convenience.

**Figure 2:**
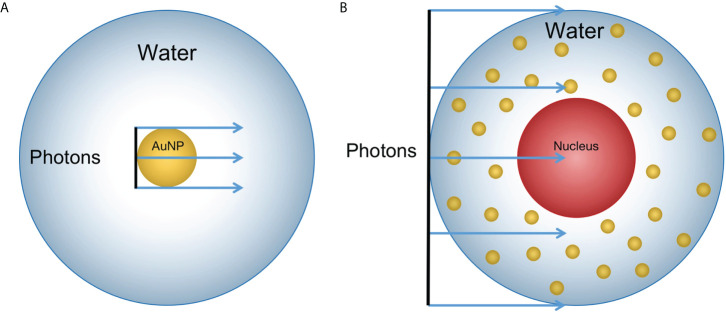
Geometry sketch of MC simulation (not the actual scale): **(A)** A 100 nm diameter AuNP was placed in a 20 μm diameter sphere filled with water. A photon beam with the same size as the AuNP in diameter was placed upstream to the AuNP. The dose was scored in the sphere shells with a thickness of 1 nm for 0 to 150 nm; 10 nm for 150 nm to 1.95 μm; and 100 nm for 1.95 μm to 9.95 μm from the AuNP surface. **(B)** The 10 μm diameter water sphere contained a 5 μm water sphere in the center to model the cell containing a nucleus. The 100 nm diameter NPs were randomly distributed in the cytoplasm. The photon beam had the same diameter as the cell and was placed upstream to the cell.

The two aforementioned methods are aimed at a single NP and a single cell respectively. The two-step method can be used to analyze the electron spectra from the surface of a single NP, while a single cell includes the physical interaction between the primary beam and MNPs. Besides, both the Penelope and Livermore physics lists can be used in the interaction between particle source and MNPs (33). Therefore, in the simulation study of Fe_3_O_4_@AuNPs, we considered the calculation results of both the two-step and one-step methods to evaluate the sensitivity enhancement performance in a single NP as well as in a cell. To compare the two-step and one-step methods, we modeled the same geometry to simulate the interaction process of the photons and AuNP in water, as illustrated in **Figure 2A**, and we chose the Livermore physics list since its low energy limit (10 eV) is lower than Penelope’s (100 eV) (33).

For the two-step method, we divided the simulation into two steps, as described above. In the first step, the phase space of output electrons was obtained from the AuNP surface after being irradiated by a 50 keV photon beam within a box (200*200*200 nm^3^) filled with water. In the second step, the electron phase space was used as a particle source and placed in the center of the water sphere (20 μm diameter). The deposited dose was scored in the sphere shells from 0 to 150 nm, 150 nm to 1.95 μm, and 1.95 μm to 9.95 μm from the AuNP surface with different precisions. We set the Livermore physics list for the first step and the Geant4-DNA physics list for the second step. The de-excitation and Auger was activated to include Auger production and particle induced X-ray emission.

In the one-step method, the AuNP was placed in the center of the water box (20 μm diameter). Thereafter, the 50 keV photon beam interacted with the AuNP and the dose was recorded at the sphere shells with different distances from the AuNP surface. The AuNP region was assigned with Livermore physics list, whereas all of the other regions were set with Geant4-DNA physics list. In both methods, we recorded the dose distribution that was produced by the electrons.

### 2.3 Photon energy dependence of single Fe_3_O_4_@AuNP dose enhancement using the two-step method

We used an Fe_3_O_4_@AuNP with the same size and composition as in Hu’s test in our simulation. Five monoenergetic photon beams (50, 100, 150, 200, and 250 keV) were used as particle sources to irradiate the single Fe_3_O_4_@AuNP, AuNP, and water nanoparticle (WNP). The photon source was plane parallel with a 100 nm diameter and started at the NP surface, as illustrated in **Figure 2A**. To evaluate the properties of the Fe_3_O_4_@AuNP at different photon energies, we compared the DEFs and electron spectra from the surface (with or without the Auger process) of the Fe_3_O_4_@AuNP and AuNP that were irradiated by the same five monoenergetic photon beams with the same simulation parameters.

### 2.4 Photon energy and nanoparticle concentration dependence of cell dose enhancement using one-step method

The radiation processes were implemented in a simplified cell model. The 10 μm diameter cell contained a 5 μm diameter nucleus in the center and the cell was placed in a water box. Both the cytoplasm and nucleus were filled with water to model the cellular environment. The monoenergetic photon source (50, 100, 150, 200, and 250 keV) was plane parallel with a 10 μm diameter and started from the cell surface, as illustrated in **Figure 2B**. Considering that NPs are predominantly dispersed in the cytoplasm when NPs enter the cell (34), the 100 nm diameter Fe_3_O_4_@AuNPs and 100 nm diameter AuNPs were randomly placed in the cytoplasm in the simulation to draw a comparison.

Scientists have shown that magnetic targeting is a promising technology among passive tumor accumulation in radiotherapy. Magnetic NPs can be focused on the tumors under the magnetic field outside the body (35). However, the magnetic targeting property for magnetic material in a magnetic field cannot be simulated with the MC method. Therefore, we used different concentrations of Fe_3_O_4_@AuNPs to simulate the targeting focus of the Fe_3_O_4_@AuNPs in different magnetic field strengths. To cover the desired dose range on the cell level, the NPs mass concentration was incremented in the range of 1 to 50 mg/mL (36). Subsequently, we selected 5, 10, and 50 mg/mL as the concentration weights of the Fe_3_O_4_@AuNPs and AuNPs in the cytoplasm. The corresponding NPs numbers are listed in **Table 1**.

**Table 1:** Number of 100 nm diameter Fe_3_O_4_@AuNPs and AuNPs in cytoplasm for five concentration weights (units: mg/mL).

			
Mass/volume (mg/mL)	5	10	50
Number of Fe_3_O_4_@AuNPs in cytoplasm	307	615	3074
Number of AuNPs in cytoplasm	259	518	2588

### 2.5 Magnetic field dependence of single nanoparticle and cell dose enhancement

With the increasing use of MRI-guided radiotherapy, it is necessary to investigate the influence of the magnetic field on radiotherapy. The *in vitro* tests performed by Hu et al. demonstrated that core-shell Fe_3_O_4_@AuNPs can be used to decrease the viability of HeLa cells by improving their internalization by the cells in an external magnetic field (0.2 T) (30). Bug et al. and Lazarakis et al. demonstrated that the magnetic field affected the charged particle trajectory only (37, 38); the physical cross section, DNA strand breaks, and cluster size distribution could not be changed by the magnetic field in Geant4.

We investigated the influence of the changed particle trajectory under the magnetic field on the sensitization process of the Fe_3_O_4_@AuNP and AuNP. The simulation was performed on a single NP and a cell model using the two-step and one-step methods, with irradiation by a 50 keV monoenergetic photon beam. The NP and cell models used were the same as those described in Sections 2.3, 2.4. The diameter of the photon beam was twice the diameter used in Sections 2.3, 2.4, i.e., 200 nm and 20 μm were used in the single NP and a cell models, respectively. An external dipole magnetic field with a strength of 0.1 to 10 T was placed perpendicular to the incident particle direction in the simulations. The NPs concentration was 50 mg/mL in the cell model.

### 2.6 DEFs of Fe_3_O_4_@AuNP and AuNP interacted with brachytherapy source

**Figure 3:**
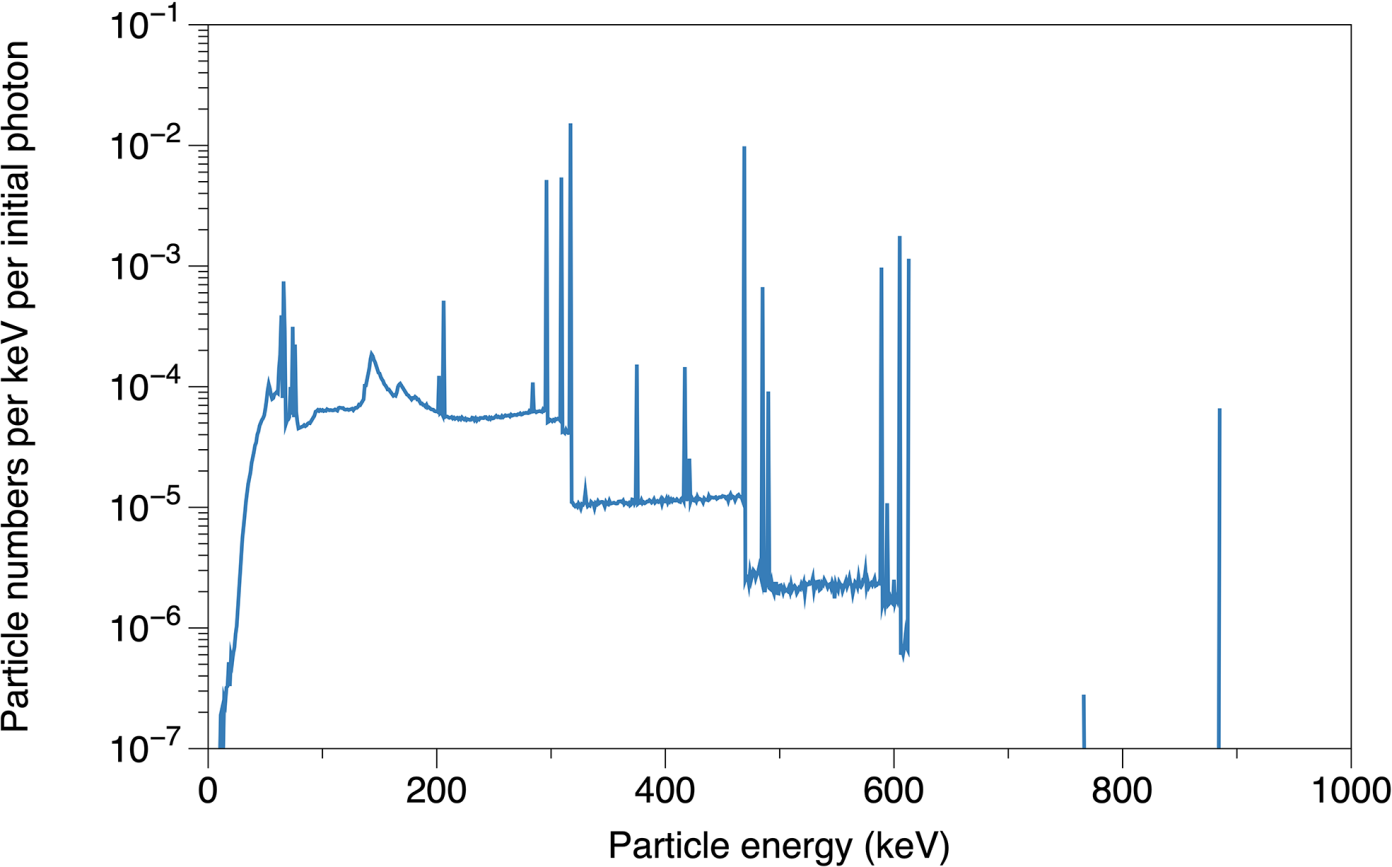
The simulated particle energy spectrum emitted from brachytherapy source model, recorded on parallel plane at 2 cm distance from source center.

In the *in vitro* tests of Hu et al. (30), the HeLa cells were irradiated by photons from a Varian linear accelerator (True Beam) with and without a dipole magnetic field (0.2 T). In this study, we further evaluated the sensitization properties of the Fe_3_O_4_@AuNP and AuNP under a clinically applied source. We implemented the Varian GammaMed Plus HDR ^192^Ir brachytherapy source model (developed by Wu et al. using TOPAS (39)) to explore the DEF of the Fe_3_O_4_@AuNP and AuNP irradiated by a brachytherapy source. The particle numbers that were emitted from the brachytherapy source model per keV per initial photon (a total of 10^8^ initial photons were used as the source in this simulation), were recorded on a parallel plane at a 2 cm distance from the source center, as presented in **Figure 3**.

The DEFs of single Fe_3_O_4_@AuNP and single AuNP were calculated using the brachytherapy spectrum, all the other parameters were consistent with those described in Section 2.3. To compare the DEFs of Fe_3_O_4_@AuNPs (with and without magnetic field) and AuNPs in the cell model, we set 50 mg/mL concentration weights for both Fe_3_O_4_@AuNPs and AuNPs ignoring magnetic field, and 1.48 × 50 mg/mL for targeted Fe_3_O_4_@AuNPs to simulate the magnetic focusing property(according to the ratio of fluorescence intensity in Hu’s tests (30)) under the magnetic field. The remaining settings were identical to those specified in Section 2.4.

## 3 Results

### 3.1 Comparison of two-step and one-step methods

**Figure 4:**
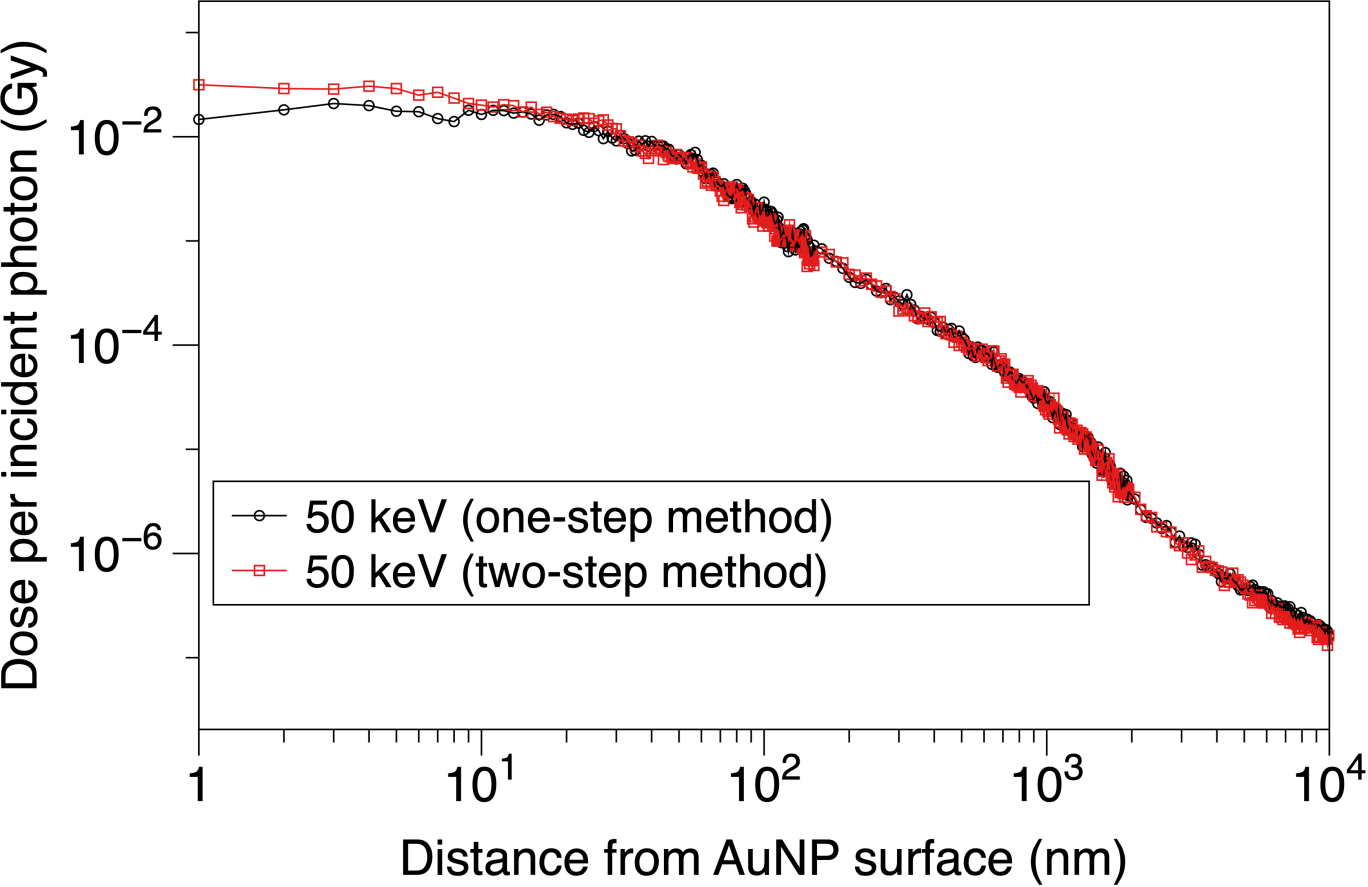
Dose distribution per incident photon vs. distance from AuNP surface.

**Figure 4** presents the results of the comparison between the two-step and one-step methods. It is obvious that the two curves show highly similar trends above 10 nm and exhibit divergence within 10 nm. Within 10 nm, the dose under the two-step method is, on average, 35% higher than the dose under the one-step method. This divergence demonstrates the unsteadiness near the AuNP surface, i.e., the boundary of different physical modules. This may be caused by the different physical judgments on the boundary of the two methods. This issue requires further research to fully understand the problem. To assign the computing resource more efficiently in our research, we use the two-step method to analyze the electron spectra from the surface of a NP and calculate the DEF around a NP, and use the one-step method to calculate the DEF in a cell model.

### 3.2 Photon energy dependence of single Fe_3_O_4_@AuNP dose enhancement

**Figure 5:**
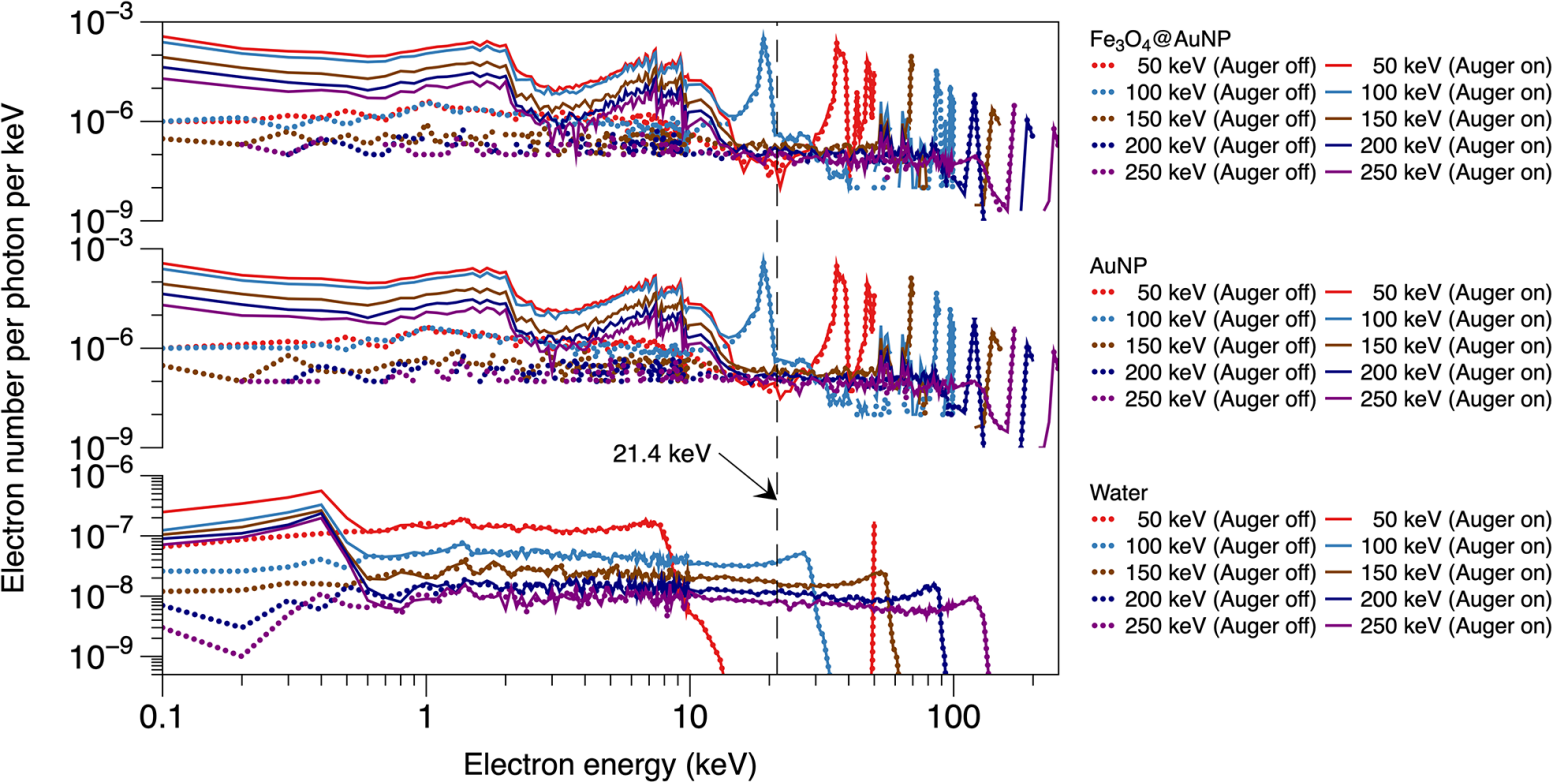
Electron energy spectra on the surface of Fe_3_O_4_@AuNP and AuNP for photon beams with various energy, with or without the Auger process.

**Figure 6:**
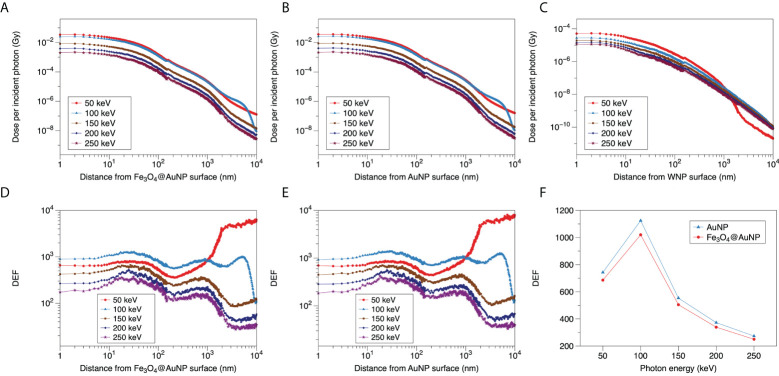
Relationship between dose distribution per incident photon and distance from surface of **(A)** Fe_3_O_4_@AuNP, **(B)** AuNP, and **(C)** WNP for 50, 100, 150, 200, and 250 keV photons. DEF distributions around **(D)** Fe_3_O_4_@AuNP and **(E)** AuNP as function of distance from the nanoparticle surface. **(F)** Total DEF around Fe_3_O_4_@AuNP and AuNP in range of 1 to 9.95 × 10^3^ nm vs. photon energy.

The results of the photon irradiations are depicted in **Figures 5**, **6**. **Figure 5** shows the electron spectra from the surface of AuNP, Fe_3_O_4_@AuNP and WNP with or without the Auger process. The energy of Auger electrons is mainly within 14 keV for Fe_3_O_4_@AuNP and AuNP, and within 1 keV for water, with higher energy electrons mainly contributed by the photoelectric process. The electron spectra of both NPs present similar wave trends for the same energy, while the total electron number of AuNP is, on average, 16% higher than that of Fe_3_O_4_@AuNP due to a higher gold content of AuNP. **Figures 6A–C** present the dose distributions at different distances from the surface of the single Fe_3_O_4_@AuNP, single AuNP, and single WNP, respectively, per incident photon. It is clear that the five dose distribution curves in both **Figures 6A, B** exhibited similar trends due to the similarity of the electron spectra. Higher energy photons caused a lower dose distribution in the energy range from 150 to 250 keV. However, the deposited dose of 100 keV photon was higher than 50 keV at the range tail (1.4 × 10^3^ to 6.8 × 10^3^ nm), owing to the photoelectric peak (at 19 keV, in **Figure 5**) of 100 keV photons near the electron range edge (9.95 × 10^3^ nm, in **Figure 6**) in water, corresponding to energy at 21.4 keV (as shown by the black dotted line in **Figure 5**, which can be described by (40)).

The DEFs of the Fe_3_O_4_@AuNP and AuNP were calculated based on **Figures 6A–C** and the results were plotted in **Figures 6D, E**. The five curves in **Figures 6D, E** also exhibited similar trends. The DEFs of both NPs receded with an increase in the photon energy for the 150, 200, and 250 keV photons. According to **Figure 6C**, the red line (50 keV photons) decreased sharply at 1 × 10^3^ nm, based on the electron energy from WNP ends at 13 keV (in **Figure 5**). However, the blue line (100 keV photons) crossed the red line at 1 × 10^3^ nm because its electron energy ending beyond 13 keV. To compare the total dose deposition in the range of 1 to 9.95 × 10^3^ nm intuitively, the doses that were distributed at different distances were totaled for each photon energy, as illustrated in **Figure 6F**. According to the figure, the DEF of the Fe_3_O_4_@AuNP was 8.7% lower than that of the AuNP on average. Moreover, the peak of the DEF versus photon energy curve appeared at 100 keV. The DEF was greatest near 100 keV in the photon energy range of 50 to 250 keV, indicating that the most sensitive energy was around 100 keV.

### 3.3 Photon energy and nanoparticle concentration dependence of cell dose enhancement

**Figure 7:**
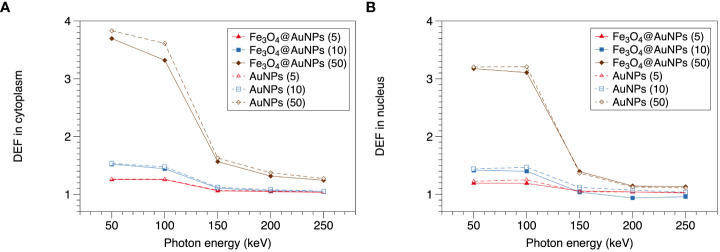
Function of DEF and photon energy in **(A)** cytoplasm and **(B)** nucleus.

The DEFs of the Fe_3_O_4_@AuNPs and AuNPs in the cytoplasm and nucleus are presented in **Figures 7A, B**, respectively. It can be observed that the 50 to 100 keV energy photons show higher DEFs than 150 to 250 keV obviously and the curves tend to flat in the range of 150 to 250 keV, both in cytoplasm and nucleus. Then it can be obtained that the sensitive energy range for cell is within 100 keV. Besides, both NPs exhibit higher DEF in cytoplasm than nucleus, meaning the sensitization of the nucleus was less than that of the cytoplasm because some low energy electrons cannot reach the nucleus.

In cytoplasm and nucleus, the DEF of the AuNPs were higher than Fe_3_O_4_@AuNPs within 100 keV. However, the differences were not obvious between 150-250 keV. In the cytoplasm, the DEFs of the Fe_3_O_4_@AuNPs and AuNPs decreased with an increase in the photon energy. The maximum DEFs of the Fe_3_O_4_@AuNPs and AuNPs for 50 mg/mL were 3.69 and 3.83. The maximum difference was within 1%, 2.2%, and 8.1% when comparing the DEFs of the AuNPs and the Fe_3_O_4_@AuNPs for the 5, 10, and 50 mg/mL NPs concentrations, respectively. In the nucleus, the maximum DEFs of Fe_3_O_4_@AuNPs and AuNPs for 50 mg/mL were 3.18 and 3.21. The maximum difference was within 5%, 13%, and 3.1% when comparing the AuNPs and Fe_3_O_4_@AuNPs for the 5, 10, and 50 mg/mL NP concentrations, respectively.

Furthermore, a higher NPs concentration led to a higher DEF in the cytoplasm and nucleus within the sensitive energy range. This means that the high magnetic focus property can achieve better dose enhancement for radiotherapy.

### 3.4 Magnetic field dependence of dose enhancement

**Figure 8:**
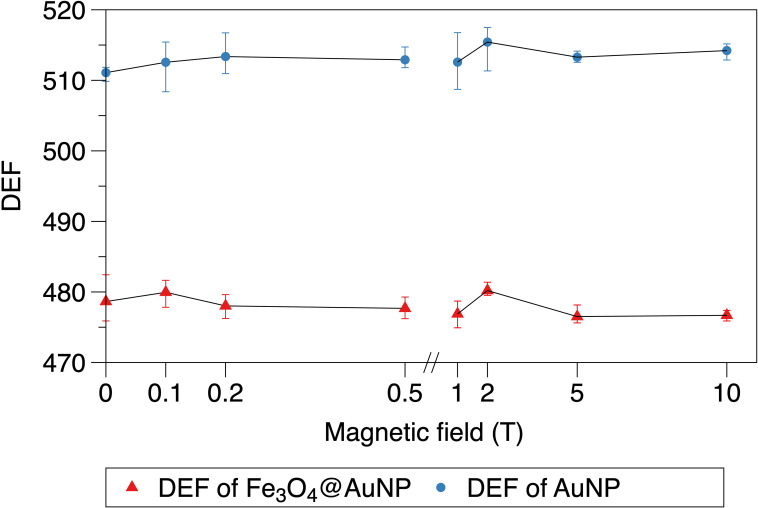
Relationship between magnetic field and DEF of a single Fe_3_O_4_@AuNP and AuNP.

**Figure 9:**
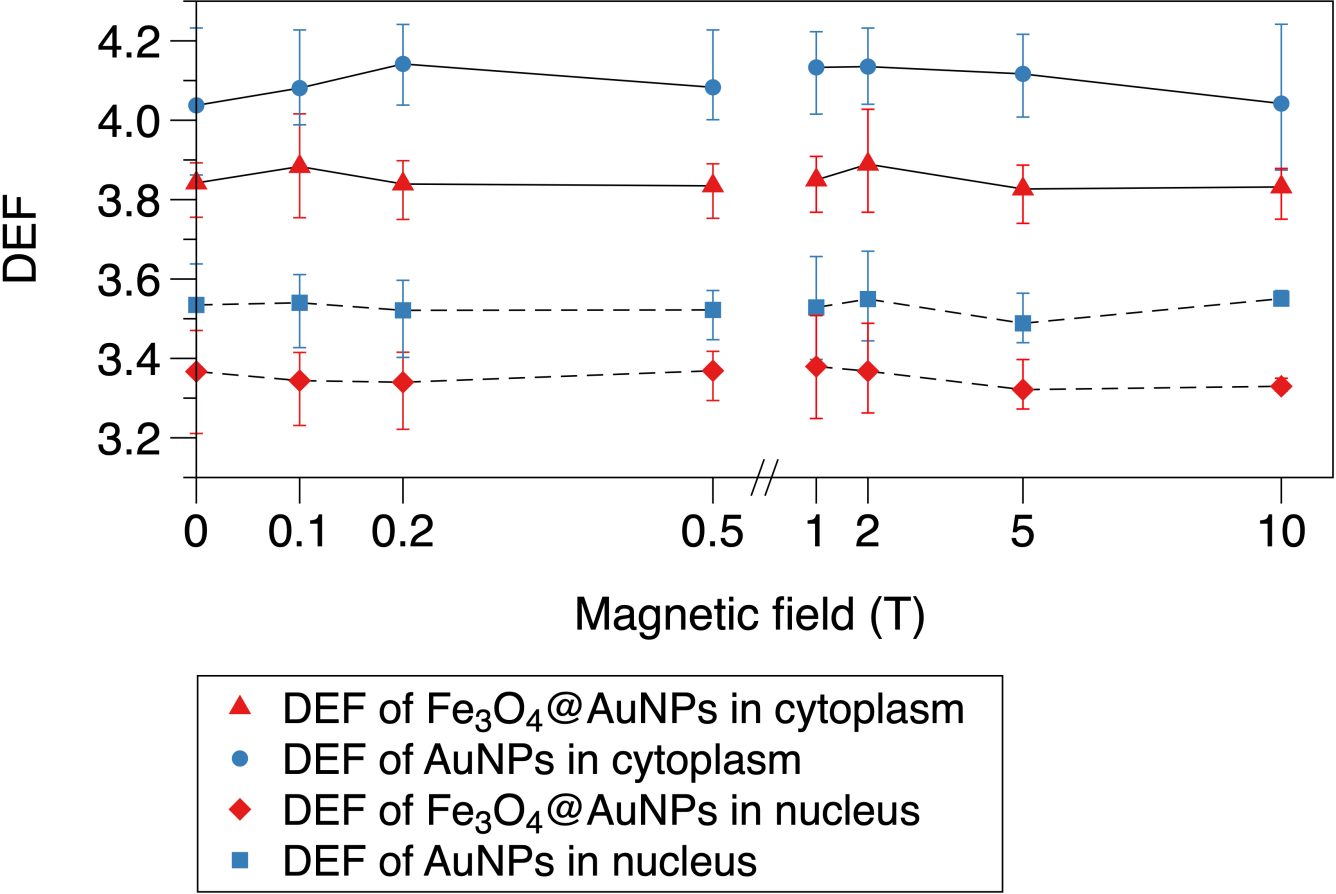
Relationship between magnetic field and DEF of Fe_3_O_4_@AuNPs and AuNPs in cytoplasm and nucleus.

The relationship between the magnetic field and DEF of the NPs is presented in **Figures 8**, **9**. We used the two-step method on a single NP and found that the DEF of the Fe_3_O_4_@AuNP was 6.7% lower than that of the AuNP under the magnetic field, as illustrated in **Figure 8**. The DEF exhibited irregular fluctuations in the range of 0 T to 10 T, with the fluctuation seemingly caused by the statistical error. This simulation result indicates that the magnetic field did not contribute significantly to the DEF.

The DEFs of the Fe_3_O_4_@AuNP and AuNP in the cytoplasm and nucleus are illustrated in **Figure 9**. We used the one-step method in a cell to simulate the influence of the magnetic field on the DEF. The Fe_3_O_4_@AuNP DEF was 6% and 5% lower than that of the AuNP in the cytoplasm and nucleus, respectively. The DEF in the nucleus was 12.9% and 13.8% lower than that in the cytoplasm for the Fe_3_O_4_@AuNP and AuNP, respectively. In general, the magnetic field did not contribute significantly to the DEF in the cell model. In this study, we concluded that a magnetic field with a strength of 0.1 to 10 T would not have a negative effect on the sensitization process, and the dose changes were mainly contributed by the magnetic targeting property under different magnetic field strength.

### 3.5 DEFs of Fe_3_O_4_@AuNP and AuNP irradiated by brachytherapy source

**Figure 10:**
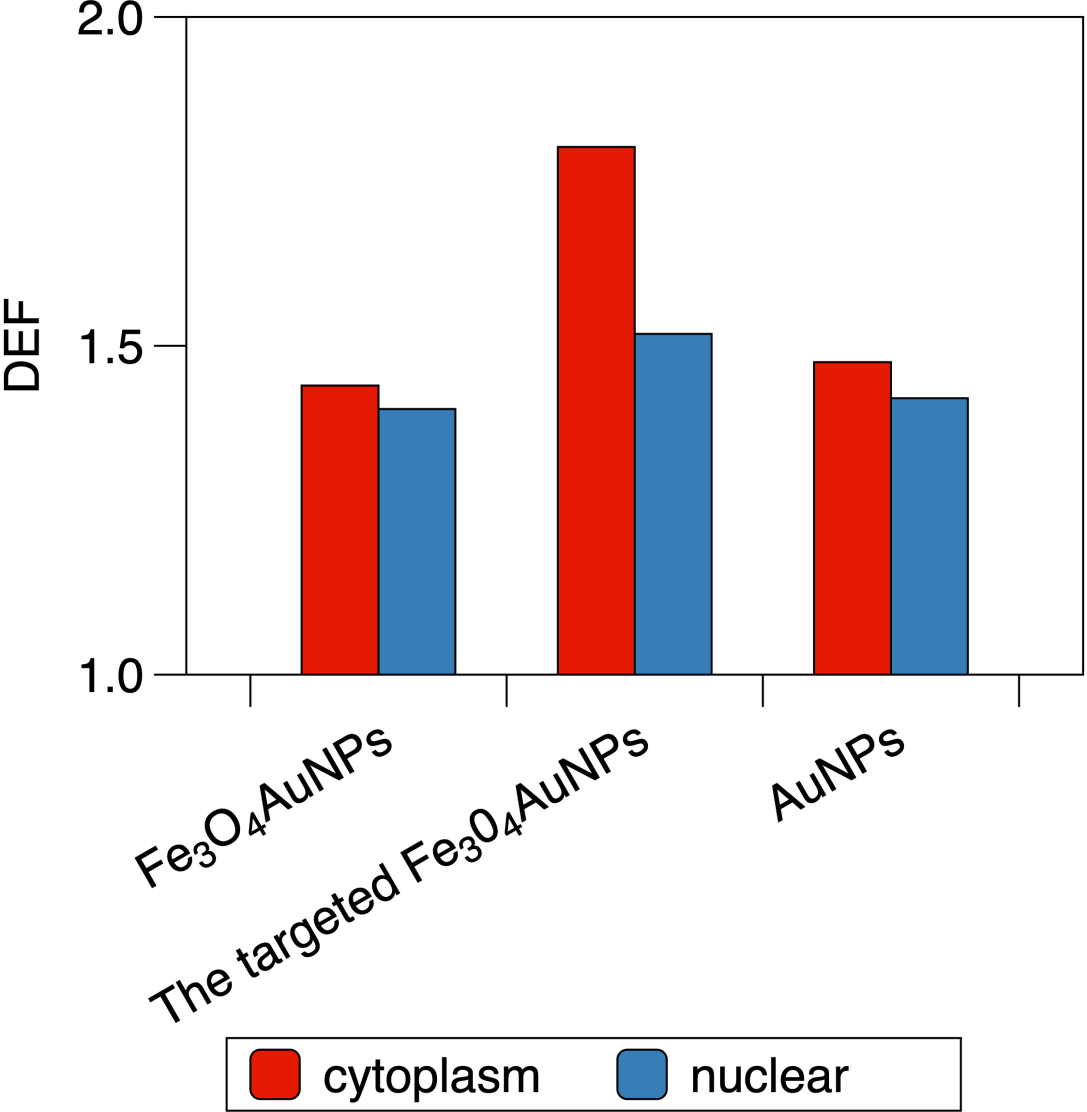
The DEF of Fe_3_O_4_@AuNP, the targeted Fe_3_O_4_@AuNP and AuNP in the cytoplasm and the nucleus when irradiated by brachytherapy source.

For the single NP model, the DEF of the Fe_3_O_4_@AuNP was 3.8% lower than that of the AuNP. For the cell model in **Figure 10**, the DEFs of the Fe_3_O_4_@AuNPs (50 mg/mL) were 2.41% and 1.15% lower than those of the AuNPs (50 mg/mL) in the cytoplasm and nucleus, respectively. However, the DEFs of the targeted Fe_3_O_4_@AuNPs (74 mg/mL) were 25.2% and 8.13% higher than those of the normal Fe_3_O_4_@AuNPs (50 mg/mL), hence 22.17% and 6.89% higher than those of AuNPs (50 mg/mL). The results revealed that despite the DEFs of Fe_3_O_4_@AuNPs being lower than those of AuNPs at the same concentration weight, the DEFs of targeted Fe_3_O_4_@AuNPs (the initial concentration was the same as that of AuNPs, and the targeted concentration was set based on Hu et al. (30)) were higher than those of AuNPs under the magnetic field. The Fe_3_O_4_@AuNPs exhibited great sensitization properties under the magnetic field.

Furthermore, the DEFs of the Fe_3_O_4_@AuNPs, targeted Fe_3_O_4_@AuNPs and AuNPs in the cytoplasm were 2.54%, 18.72% and 3.86% higher, respectively, than those in the nucleus, showing the better sensitization in cytoplasm than nucleus under the brachytherapy source.

## 4 Discussion

AuNPs are studied extensively in radiosensitization owing to their properties of high X-ray absorption, hypotoxicity, and easy synthesis. Magnetite can be used as targeting material to improve tumor drug delivery because of the magnetic targeting property in the magnetic field (35). As a novel NP, the Fe_3_O_4_@AuNP combines the properties of gold and magnetite, and it has been used in *in vitro* experiments to decrease the cell survival rate (30).

In this study, we compared the one-step and two-step methods for calculating the DEF in an AuNP model. There was a 35% discrepancy between the two methods within a 10 nm distance from the AuNP surface, which may be due to the different physical judgments on the boundary. However, no significant difference was observed between the two methods in the range of 10 to 9.95 × 10^3^ nm distance from the AuNP surface. We selected the two-step method for a single NP to analyze the electron spectra, and the one-step method for a cell.

We explored the DEF of an Fe_3_O_4_@AuNP in a single NP and in a cell model compared to the AuNP. The DEF around the single Fe_3_O_4_@AuNP was 8.7% lower than that of the AuNP, and the differences between the AuNP and Fe_3_O_4_@AuNP in the cytoplasm and nucleus are detailed in **Figure 7**. **Figure 7** shows that a higher NPs concentration resulted in a higher DEF, proving the magnetic targeting property led to a better dose enhancement. The sensitive energy range for a NP and cell was obtained as within 100 keV. It was expected that the DEF of the Fe_3_O_4_@AuNP would be lower than that of the AuNP within the sensitive energy because the photoelectric cross section of iron and oxygen is lower than that of gold. We quantified the discrepancy between the Fe_3_O_4_@AuNP and AuNP to provide an analysis of core-shell magnetic NPs that are used as sensitivity materials. It is well known that the clustering property of AuNPs will decrease the DEF in a cell in radiotherapy. However, there were no obvious Fe_3_O_4_@AuNP clusters observed in Hu’s test under the magnetic field (30). This also demonstrates the superiority of Fe_3_O_4_@AuNP over AuNPs in radiotherapy combined with magnetic field. Significant analytical potential exists for decreasing the cluster by using the magnetic NPs under an extra magnetic field, so as to increase the DEF.

We investigated the influence of the magnetic field on the DEF and demonstrated that the magnetic field did not have a significant effect on the sensitization process. The results revealed that the changed electron trajectory was insufficient to influence the dose enhancement, or the electron trajectory was insufficient to be changed with such electron energy and the magnetic field (37). Therefore, the physical enhancement was not degraded by the magnetic field due to its small amplitudes relative to electron energy. Combined with the *in vitro* experiment carried out by Hu, we verified that the radiosensitization mainly benefited from the physical enhancement of Fe_3_O_4_@AuNP in addition to the magnetic focusing property combined with magnetic field.

Furthermore, we constructed a brachytherapy source for irradiation with a single NP and a cell model. The results of the brachytherapy irradiation showed the residuals between the Fe_3_O_4_@AuNP and AuNP in a single NP and a cell model. The DEF of Fe_3_O_4_@AuNP was 3.8% lower than AuNP for a single NP. In the cell model (50 mg/mL NPs), the DEFs of Fe_3_O_4_@AuNP were 2.41% (cytoplasm) and 1.15% (nucleus) lower than AuNPs, while the targeted Fe_3_O_4_@AuNP enhanced the DEFs and 22.17% and 6.89% higher than AuNPs under the magnetic field. The results clarified the superiority of the Fe_3_O_4_@AuNP combined with a magnetic field under the brachytherapy source. The DEFs in cytoplasm were higher than that of the nucleus no matter under brachytherapy or monoenergetic photons, showing that different regions in a cell exhibit different sensitization properties. The results clarified the dose enhancement of the Fe_3_O_4_@AuNPs under the brachytherapy source.

The study is based on a cell model, however, it is more challenging to define the distribution of NPs in tissues and there is still a long way to go from the cellular scale to the tissue scale. In the future, research on guiding the Fe_3_O_4_@AuNPs to focus on tumors through the magnetic field will be quite beneficial due to the development of MRI-guided radiotherapy (41). For example, the source applicator may be magnetized to guide magnetic NPs or the sensitization may be combined with MRI-guided brachytherapy to focus the magnetic NPs. This research may raise concerns regarding MRI-guided brachytherapy combined with magnetic NPs.

## 5 Conclusions

In this work, we compared the one-step and two-step methods for calculating the DEF. Then we applied the two methods to a single particle and a cell model to investigate the DEFs of the Fe_3_O_4_@AuNP and AuNP. The DEF of the Fe_3_O_4_@AuNP was 8.7% lower than that of the AuNP in a single particle. In the cell model, the DEF difference between the Fe_3_O_4_@AuNP and AuNP was below 8.1% in the cytoplasm with an NPs concentration of 5 to 50 mg/mL, and the targeting property contributed to the dose enhancement. We also demonstrated that the magnetic field has no detrimental effect on the NPs radiosensitization. Furthermore, we applied a brachytherapy source for interaction with the Fe_3_O_4_@AuNP and AuNP in a single NP and a cell model to obtain the DEF in brachytherapy source irradiation, and proved the DEF of Fe_3_O_4_@AuNP targeted by magnetic field exceeded the AuNP with the same concentration weight.

In summary, this study revealed the Fe_3_O_4_@AuNP properties systematically in radiotherapy dose enhancement using the MC method for the first time. Moreover, we demonstrated that the physical dose enhancement of the Fe_3_O_4_@AuNP is independent of the magnetic field. Finally, we determined the DEF of Fe_3_O_4_@AuNPs in a brachytherapy source to provide simulation results for future clinical research and demonstrate the significant potential of using Fe_3_O_4_@AuNPs to enhance dose deposition combined with a magnetic field. In future research, Fe_3_O_4_@AuNPs may be combined with a magnetic field (such as MRI) to overcome the issue of NPs clustering and to improve the NPs concentration in cells. This will be desirable for future *in vitro* tests on radiosensitization as well as clinical research.

## Data availability statement

The raw data supporting the conclusions of this article will be made available by the authors, without undue reservation.

## Author contributions

XX contributed to conceptualization, methodology, validation, formal analysis, investigation, resources, data curation, original draft preparation, review and editing. JW contributed to conceptualization, software, validation, review and editing. ZD contributed to conceptualization, review and editing. RH contributed to data curation. YX contributed to supervision and project administration. LW contributed to supervision and funding acquisition. All authors contributed to the article and approved the submitted version.

## Funding

This work was supported by the Sanming Project of Medicine in Shenzhen (No. SZSM201612063), Shenzhen High-level Hospital Construction Fund, and National Natural Science Foundation of China (No. 12105367).

## Conflict of interest

The authors declare that the research was conducted in the absence of any commercial or financial relationships that could be construed as a potential conflict of interest.

## Publisher’s note

All claims expressed in this article are solely those of the authors and do not necessarily represent those of their affiliated organizations, or those of the publisher, the editors and the reviewers. Any product that may be evaluated in this article, or claim that may be made by its manufacturer, is not guaranteed or endorsed by the publisher.
